# Dignity violation of patients with schizophrenia: a grounded theory

**DOI:** 10.1186/s12888-025-07472-1

**Published:** 2025-10-22

**Authors:** Elham Amiri, Rahim Baghaei, Hossein Habibzadeh, Hossein Ebrahimi

**Affiliations:** 1 faculty of Nursing and Midwifery, Urmia University of Medical Sciences, Urmia, Iran; 2 faculty of Nursing and Midwifery, Tabriz University of Medical Sciences, Tabriz, Iran

**Keywords:** Grounded theory, Dignity, Schizophrenia, Patient

## Abstract

**Background:**

Considering that dignity is a fundamental entitlement of each patient, respecting the dignity of patients is essential. Unfortunately, in many cases, especially among people with schizophrenia, dignity is not fully respected, which can challenge their coping strategies, and affect the results. Nonetheless, there is limited knowledge regarding this matter in Middle Eastern Nations.

**Objectives:**

Investigating the process of dignity violation in patients with schizophrenia.

**Methodology:**

The present study data were collected through field notes, semi-structured, face-to-face interviews with 16 patients with schizophrenia (4 patients had partial insight, and 12 patients had complete insight), 4 family caregivers, 2 nurses, 3 psychologists, and 1 service worker with maximum variation. The data were analyzed through the consistent comparison methodology of Grounded Theory, and coded using MAXQDA-10 software.

**Results:**

Data analysis showed that the main concern of the patients was “Being ignored by others”. The contextual factors contributing to “Being ignored by others” were “Being ignored by family”, “Silent treatment organization”, and “Sociocultural barriers”. The patients used the main strategy of “Attempting to violate mutual dignity” to deal with their main concern. This strategy led to some consequences that have been examined in a main category of “Death wish”. Overall, the efforts that the patients used to protest the threat to their dignity were not effective, and further exposed them to threats to their dignity. The category of “Death wish” has two subcategories: “Patient’s internal dissatisfaction”, and “Dependency”.

**Conclusion:**

The results of the present study showed that maintaining the dignity of patients with schizophrenia is a downward cycle due to the negative mentality of those around them. Violation of the basic rights of patients by their families, social exclusion, and failure to act according to instructions are common in psychiatric wards. However, the results of this study showed that patients strive to maintain, and regain their dignity even in the most difficult circumstances. Psychiatric nurses can empower patients with the essential abilities to counteract dignity violation, and modify the sense of disability as an obstacle to dignity violation resistance.

## Background

Human dignity as an ethical value is a fundamental notion in the field of medical ethics [1]. There is a lack of consensus among theorists regarding the position, and utility of dignity as a notion in the field of bioethics with certain proponents contending that dignity is essentially just respect for people, and their autonomy [2, 3]. However, others argue that dignity is a concept in its own right, and is particularly important for people with limited autonomy [4, 5].

Dignity in the context of healthcare is when a person has autonomy and the ability to make decisions and exert control over their environment, their behavior, and how others interact with them [6]. Since autonomy acknowledges a person’s competence, Undermining patients’ abilities diminishes their character and self-esteem, turning them into helpless and passive recipients of care [7]. On the other hand, dignity is fostered when a healthcare service user is given the skills and authority to make educated choices and provide informed consent for their care. Leveling asymmetries of power within the therapeutic relationship is essential to empowering patients’ autonomy and dignity by giving them information and tools to make choices about their care [8, 9].

Dignity is reciprocal. People treat you with respect when you conduct yourself in a dignified way. Similarly, a person’s dignity is reduced when they are treated with disrespect [10]. Furthermore, Jackson & Irwin (2011) emphasizes that dignified behavior is essential throughout the caregiving process, even after death, and that when nurses treat patients with respect, it leads to feelings of worth, utility, trust, and autonomy in decision-making [11]. In conclusion, dignity-centered care enhances the caliber of treatment given and fosters mutual trust, comprehension, and open communication between patients and caregivers by upholding their shared dignity [12].

Dignity preservation is characterized by respect, individualized care, restoration of defensive control, and active listening; dignity conservation in care has been described as a continuity of oneself, role maintenance, hope preservation, pride, autonomy, acceptance, resilience, and spiritual struggle [13, 14]. Griffin-Heslin identified the attributes, and related attributes of dignity as Respect, Autonomy, Empowerment, and Communication [15].

Dignity violation is frequently observed when one party is in a state of vulnerability, while the other party is in a position of antipathy [16] Health problems, and illnesses have been proven to be among the main factors that seriously threaten dignity [1]. This concern assumes even greater significance in the case of patients with psychiatric disorders, owing to their inherent vulnerability, and specific medical conditions [17]. It is therefore widely accepted that schizophrenia is one of the most disabling diseases. Frequent hospitalizations, and treatment costs are the main features of this disease, which is characterized by a variety of complications [18]. While the United Nations Convention on the Rights of Persons with Disabilities (CRPD) emphasizes that these patients are entitled to full access to all rights and equal opportunities [19], in fact, particularly in low-income countries, they have limited access to health services and they are excluded from community-based rehabilitation programs [20]. The problem is that there is little legislative action at national level to ensure the right of patients with mental illness to adequate social support and protection from discrimination [21]. A violation of patient rights could therefore lead to a violation of human dignity [22].

Violation of human dignity can affect patients’ bodies, minds, moods, morals, and spirituality, exposing them to stress and discomfort. A lack of respect for human dignity leaves patients feeling unsafe, humiliated, and ashamed, which can negatively impact treatment outcomes and prolong hospital stays [23]. The people around a patient can greatly influence his or her sense of dignity. Numerous studies distinguish the factors that promote or undermine patient dignity [24], and many of these are related to the patient’s environment. Factors that can enhance a hospital patient’s sense of dignity include treating the patient as a holistic person [25–27], satisfactory communication [28–30], and respect [25, 26, 29, 30], However, a hospital patient’s dignity can be undermined by being treated as an object [25, 27], poor communication [26, 30], and inappropriate actions by medical staff [27, 28, 30].

According to our literature review, there are three significant shortcomings in the field, which are addressed in this study: (A) the use of descriptive and phenomenological research methodologies, which do not lend themselves to the creation of a theoretical explanation of the recovery process; (B) a limited variety of data sources and a lack of research that incorporates input from other people who are directly concerned with the recovery (people living with schizophrenia, family members, and health professionals); and (C) a scarcity of nursing research in the area that would take into account interactions among all biopsychosocial dimensions. On the other hand, the role of addressing dignity violations in patients with schizophrenia in determining their vulnerability to this phenomenon may have a significant impact. The process encompasses the various strategies implemented by patients to address dignity violations, the factors that influence the adoption of these strategies, and the consequences of the strategies. Therefore, the researcher decided to clarify the dimensions of the phenomenon by conducting an in-depth investigation. Certainly, identifying the background and dimensions of the violation phenomenon will help remove obstacles to maintaining the dignity of patients with schizophrenia. Hence, this study was conducted to elucidate the process of addressing the dignity violation process in patients with schizophrenia within the cultural context of Iran.

### Aim

The purpose of this study is to propose a theoretical explanation for the violation of dignity in patients with schizophrenia from the perspectives of patients, healthcare personnel, and family caregivers. This research is qualitative in nature and aims to answer the following question:

How do patients, healthcare personnel, and family caregivers describe the process of violating patients’ dignity?

## Methodology

The process of dignity violation in this situation was explored by adopting the theoretical approach of Corbin and Strauss. It offers novel perspectives on various experiences and phenomena. The approach elucidates the manner in which individuals present their actions, interactions, and emotions in response to events, situations, or problems, thereby contributing to a comprehensive understanding. Grounded Theory is a qualitative research methodology aimed at generating a theory that is grounded in systematically gathered and analyzed data. According to Corbin and Strauss, this approach involves iterative data collection and constant comparative analysis to identify patterns, categories, and relationships within the data [31]. Given that dignity is fostered within the context of social interactions and influenced by how individuals react to violations of dignity, employing a grounded theory approach is suitable in this particular context.

### Participants and study setting

In total, 26 participants including 16 patients with schizophrenia (4 patients had partial insight and 12 patients had complete insight), 4 family caregivers, 2 nurses, 3 psychologists, and 1 service worker were included in this study, whose demographic characteristics are presented (Table 1).

**Table 1:** Demographic characteristics of the study participants

	**Characteristics**		**n (%) **
Patients with Schizophrenia	Gender	Male	10(62.5)
		Female	6(37.5)
	Marital status	Married	3(18.75)
		Single	8(50)
		Divorced	5(31.25)
	Occupation	Unemployed	5(31.25)
		Housewife	6(37.5)
		Employee	5(31.25)
	Educational status	Primary school	6(37.5)
		High school diploma	6(37.5)
		Bachelor’s degree	4(25)
	Age		Mean ± SD 44.38±11
Family caregivers	Relationship with patient	Sister	2(50)
		Spouse	1(25)
		Mother	1(25)
	Educational status	Primary school	3(75)
		Bachelor’s degree	1(25)
	Age		Mean ± SD45.75±18
Nurses	Educational status	Master’s degree	1(50)
		Bachelor’s degree	1(50)
	Work experience		Mean ± SD 25±3.5
	Gender	Male	2(100)
		Female	0(0)
	Age		Mean ± SD 28.5±0.7
psychologist	Educational status	PhD degree	1(34)
		Bachelor’s degree	2(66)
	Work experience		Mean ± SD 12±2
	Gender	Female	3(100)
		Male	0(0)
	Age		Mean ± SD32.5±2
service worker	Educational status	High school diploma	1(100)
	Work experience		22
	Gender	Male	1(100)
		Female	0(0)
	Age		45

This study was conducted in three psychiatric hospitals, one day center, and four 24-hour mental health centers in northwestern Iran as referral centers in this region. These places had similar protocols in place, including locking front doors, scheduling food and medicine distribution, and controlling access to cigarettes. The sampling location was not limited to the sites listed, and when necessary, samples were also attempted at other locations, such as the homes and workplaces of patients, when qualified participants were available.

In addition, purposive and snowball sampling techniques were used to identify eligible participants and the sampling process continued until data saturation was reached. When selecting participants, great emphasis was placed on maximizing the diversity of demographic characteristics to obtain a wide range of experiences. Inclusion criteria for patients were: (A) diagnosis of schizophrenia based on a psychiatrist’s assessment based on the Diagnostic and Statistical Manual of Mental Disorders (DSM-5) [32], (B) partial or complete insight (the degree of the patient’s awareness and understanding about being a patient based on the information in the patient record) [33], (C) a duration of illness more than 2 years, (D) age over 18 years, no history of ECT treatment within the past 3 months, and (E) a documented medical history of at least two years.

Inclusion criteria for Family caregivers were: (A) at least 2 years of communication with the patient and involvement with his/her condition, and (B) showing interest in cooperating with the study. Inclusion criteria for healthcares were: (A) having at least one year of experience providing mental health services to patients with schizophrenia, and (B) showing interest in cooperating with the study.

### Data collection

Concurrent data collection and analysis occurred from May 2022 to January 2023. The present study data were collected through field notes, and face-to-face interviews. The interviews are a combination of unstructured and semi-structured interviews. The researcher utilized a digital voice recorder to document the interviews. The initial five interviews were unstructured in nature and featured an open-ended question: “please tell me how the people around you behaved towards you after they found out about the disorder”. After answering this question, the researcher asked: “How did you react to such behavior from others?”. Then, depending on the purpose and method of study, probing questions such as “How?”, “Can you explain more?”, “What factors affected your responses?”, and “What was the result of these responses?”. After analyzing the initial interviews, she conducted semi-structured interviews based on the concepts and propositions created in the form of theoretical sampling. Each interview lasts between 45 and 70 min. The primary participants in this grounded theory study were the patients. However, family caregivers, nurses, and psychologists were also included through theoretical sampling to enrich the emerging conceptual framework. After completing a total of 24 interviews, the researcher achieved theoretical saturation regarding the development of concepts and the consistency of relationships among them. In order to ensure that there were no additional data points pertaining to any of the aforementioned concepts, two additional interviews were carried out. An example of concept creation is shown in Table 2.

**Table 2:** An example of the formation of concepts

Main Category	Subcategories	Initial concepts	Open codes
Attempting to violate mutual dignity	Mutual disregard for others	Distance from those around you	Patient distancing from staff due to inappropriate behavior
			Distancing oneself from others due to fear of rejection
			Distance and secrecy following the loss of trust in those around
		Ignoring insults from those around you	The patient’s indifference following the family’s neglect of the patient’s needs.
			The patient’s indifference in response to inflexible behavior from the staff
			Indifference in response to neglect from those around
			Non-compliance with treatment due to unprofessional conduct of staff with the patient
	Mutual disrespect for others	Violation of the dignity of unaccountable family members	Patients arguing with their families because of their family’s irresponsibility towards them.
			Distrust in the patient towards the indifferent family
		Violation of the dignity of unaccountable personnel	Erosion of trust in the patient due to staff unresponsiveness
			Arguments initiated by the patient upon witnessing staff unresponsiveness
Death wish	Patient’s internal dissatisfaction	Psychological burnout	Severe discomfort
			Internalized stigma
			Experience of emptiness
		Unstable relationships	Family relationship: a lose-lose situation
			The harsh reaction of those around, following the patient’s protest against the family’s insult.
		Simultaneous experience of the inner resentment and reducing tension with those around you	Inner resentment from the silence following the disrespect of those around.
			Reducing tension with others by ignoring their disrespect
		Simultaneous experience of unstable relationships and reduced psychological pressure	Disruption of relationships
			Reduction of psychological pressure following protests against the disrespect of those around.
	Dependency	Distrust towards the patient	The community’s lack of trust in the patient with psychosis symptoms
			Family distrust following the patient’s exhaustion
		Instability in social roles	Inability to perform job duties
			Dropping out of school
			Restriction on marriage
			Isolation
		Vicious cycle of disease	Disease progression
			Non-compliance with treatment

Another way of collecting data in the present study was through field notes. Observations taken during field visits were recorded. The act of documenting field notes is a well-established method for capturing observations [34], and this approach is widely accepted in the field of qualitative research [35]. They are regarded as objective evidence that aids in understanding dignity violations among patients with schizophrenia. Family caregivers, patients, and healthcare professionals were briefed on the project’s objectives. To document our observations, the primary technique we utilized was writing field notes.

### Data analysis

The constant comparative analysis approach recommended by Corbin and Strauss was utilized in this study. This approach consists of five distinct phases: “Open coding”, “Developing concepts in terms of their properties and dimensions”, “Analyzing data for context”, “Bringing the process into the analysis”, and “Integrating categories” [31].

The transcripts from the interviews and observations were reviewed multiple times. The key statements were then categorized based on what the participants expressed. These categories were organized according to shared concepts. Afterward, the initial categories were compared with the codes, merging similar items. The methods employed for analyzing the data throughout the study included constant comparison (an ongoing interaction between analysis and data collection) and theoretical sensitivity (developed through memoing, which is the practice of documenting thoughts, feelings, decisions, ideas, processes, and analytical insights as they arise during data collection, coding, and analysis). We employed a constant comparative approach in which specific data points were consistently compared to other data points to establish categories and concepts [31].

The data obtained from the study were then categorized and analyzed using MAXQDA 10. In the initial phase of “Open coding”, the researcher carefully examined the interviews and identified relevant portions of the text to address the research question. These identified portions were then coded and grouped to form primary categories. Throughout this process, new meanings were recorded in memos for further exploration in subsequent interviews. The subsequent stage, referred to as “Developing concepts in terms of their properties and dimensions”, encompassed the examination of these memos by the researcher via theoretical sampling interviews. Additionally, negative cases that did not align with the data pattern were analyzed at this stage to enhance the depth of the findings. After this stage, the concepts were meticulously expanded upon in terms of their properties and dimensions. In order to ascertain the main concern, the researcher meticulously assessed the interviews, codes, and concepts with the aim of delving into the most significant issue related to dignity violation among the patients. Specifically, the researcher aimed to understand which issue prompted the study participants to adopt the majority of the strategies. The subsequent phase, “Analyzing data for context”, entailed a thorough review of the interviews to extract factors that provided the contextual background for dignity violation among the patients. The following phase, “Bringing the process into the analysis”, involved reviewing the data to uncover the strategies employed by patients to address dignity violation, as well as the intervening factors that influenced their adoption and the consequences of these strategies. Finally, the interviews, codes, and concepts were thoroughly reviewed to uncover the core category, which represented the primary strategy adopted by individuals with schizophrenia to address dignity violation. To present the theory and integrate the categories around the core concept, the researcher employed the descriptive summary memo technique. This involved reviewing the interviews to obtain an overall understanding of the data, rather than focusing on specific details. Subsequently, the researcher crafted a concise storyline of the data utilization of several explanatory sentences [31]. Based on the position of each concept relative to the core category and the types of relationships between the concepts, the researcher integrated the categories surrounding the core category. Finally, once the theory had been honed and streamlined, it was ultimately presented.

### Ethical considerations

The research underlying this article was performed in accordance with the Declaration of Helsinki. The study received ethical approval from the Research Ethics Committee of Urmia University of Medical Sciences (Ethics code no. IR.UMSU. REC.1401.099), The interviewer was introduced to the participants and explained the study objectives and methods, and informed consent was obtained from the patient and legal guardian. Thus, all participants and legal guardians of patients participating in the study received a written information sheet and consent form. The participants and legal guardians of patients were thus able to give their free and informed consent for both their participation in the study and the audio recording of the interviews. All recordings were kept under lock and key in a secure location throughout the collection and analysis of the data. The recordings will be destroyed by the researcher herself when the results are published. The participants and legal guardians were also assured of the confidentiality of the information collected by the use of pseudonyms to avoid recognition. Also, participants were entitled to withdraw from the study at any time.

### Trustworthiness of the study

To ensure the trustworthiness of the results, a set of criteria was utilized, including credibility, dependability, conformability, and transferability [36]. The continuous presence of the researcher in the field served to enhance their comprehension of the phenomenon, thereby bolstering their credibility. The allocation of adequate time for the collection and analysis of data allowed the authors to acquire profound insights into the data. In order to ensure transferability, all research processes were meticulously documented and the interviews, coding, and analysis were reported. The establishment of dependability involved the utilization of various techniques, including the agreement between the extracted categories and sub-categories through the research team’s opinions and the double-checking of the codes. Confirmability was ensured through the review of numerous interviews, extracted codes, and categories by two faculty members who were well-versed in qualitative research data analysis.

## Results

In total, 26 participants including 16 patients with schizophrenia (4 patients had partial insight and 12 patients had complete insight), 4 family caregivers, 2 nurses, 3 psychologists, and 1 service worker were included in this study. Data analysis showed that the main concern of the patients was “Being ignored by others”. The contextual factors contributing to “Being ignored by others” were “Being ignored by family”, “Silent treatment organization” and “Sociocultural barriers”. The patients used the main strategy of “Attempting to violate mutual dignity” to deal with their main concern. This strategy led to some consequences that have been examined in a main category of “Death wish”. Overall, the efforts that the patients used to protest the threat to their dignity were not effective and further exposed them to threats to their dignity. The category of “Death wish” has two subcategories: “Patient’s internal dissatisfaction” and “Dependency”.

### Main concern: being ignored by others

In the current study, the main concern of the patients was that the people around them distanced themselves from them and did not support them when they needed it. In dealing with the patient, they behave with indifference and show no desire to communicate. Negative feedback from others, such as being ignored, could infuse a sense of worthlessness in patients, which was explained by transferring a sense of worthlessness and tangential life with patients.

In this regard, one of the patients said:

“*After three years*,* I found out about my brother’s death. In these circumstances*,* I was saddened by my brother’s death and also by my family’s secrecy. Being ignored bothers me a lot.*” (Patient 1, Male, aged 40).

### Context

Data analysis showed that the main concern of the patients was “Being ignored by others”. The contextual factors contributing to “Being ignored by others” were “Being ignored by family”, “Silent treatment organization”, and “Sociocultural barriers”.

#### Being ignored by family

The family caregivers of the patients with schizophrenia had unique capacities to preserve patient dignity, varying from one family to another. Some families have special individual capacities to maintain the patient’s dignity; some families were indifferent to maintaining the dignity of their patient, which was classified in the form of “Being ignored by family”; the category of “Being ignored by family” can be explained in the form of three subcategories of “Rejection”, “The family’s tangential life with their patient”, and “Non-acceptance of the disease condition”.

One of the patients said:

“*My mother shows affection towards my siblings*,* but they are indifferent to me and I have been excluded from the family circle*.” (Patient 12, Male, aged 38).

Similarly, another patient residing in a 24-hour mental health facility shared their experience of family members neglecting their basic needs:

*“I don’t have shampoo and toothpaste. My sister brings me my things. My sister has not visited me for two months.*” (Patient 5, Female, aged 32).

One of the nurses said:

“*Because patients with chronic schizophrenia are chronic and families see that the patient is not improving*,* they reject them*.” (Nurse 2, Male, aged 34).

##### Field Note 1

I was at a psychiatric hospital when I realized that a patient who had been discharged four months earlier according to the doctor’s orders was still in the hospital, and no family member had come to pick him up. The hospital decided to send the patient home by ambulance. The family had refused to allow the patient to enter their home. The patient was readmitted to the hospital again. (Field note 1)

##### **Field note 2**

At the hospital, I witnessed the discharge of a patient with schizophrenia. The nurse tried to contact the patient’s family for discharge. Efforts were made to contact each family member, but no one came for discharge. The nurse laughed mockingly and stated that since it was discharge time, everyone seemed to have other things to do. A relative claimed to be out of the country. Another stated that they were in an important meeting and could not come for discharge. (Field Note 2)

#### Silent treatment organization

The category of Silent treatment organization can be explained in the form of two subcategories “lack of facilities” and “premature management”.

One of the participants, residing in a round-the-clock mental health facility, expressed:

*“This ward can admit and accommodate a maximum of 20–25 patients. However*,* at present*,* there are 38 patients. In terms of meals*,* we are only provided with a bowl of soup which consists solely of water and carrots. I have to eat dry bread and refrain from expressing any objections. I feel as if our humanity is not being taken into consideration.”* (Patient 3, Male, aged 43).

The study participants revealed that the monitoring of healthcare centers lacked adherence to specific criteria, with evaluations being primarily based on personal relationships rather than established rules.

A successful manager in this context commented:

*“Unfortunately*,* many violations are overlooked by supervisors due to the prevalence of personal relationships that have replaced formal rules. In this system*,* the patient has no value; they are merely breathing.”* (Psychologist 1, Female, aged 45).

#### Sociocultural barriers

Patients with schizophrenia face many cultural and social problems in maintaining their dignity. In this study, of Black Shadow of stigma category represents a group of important barriers to respecting the dignity of patients with schizophrenia, which includes two subcategories: “Fear and discrimination experience”, and “Misconceptions in society”.

One of the participants said:

*“If my illness worsens*,* my family will not come to our home. They’re afraid of me.”* (Patient 3, Female, aged 53).

The sister of one of the patients said:

*“My sister had a suitor*,* our neighbors told her suitor that my sister had become ill … the name of the illness stuck to her like a stain.”* (Family caregiver 1, Female, aged 50).

### Strategy: attempting to violate mutual dignity

In addressing “Being ignored by others” (Main concern), participants used the main strategy of “Attempting to violate mutual dignity”, which is characterized by the features of “Mutual disregard for others”, and “Mutual disrespect for others”. The formation of these negative strategies in patients has led to more negative consequences for them and has further facilitated the violation of their dignity.

#### Mutual disrespect for others

When the staff were indifferent towards the patients, the patients’ mindset towards them became negative. They rejected the treatment and expressed greater dissatisfaction with the care received. Being ignored by nurses led to an increase in patients’ voices of protest. Patients would resort to the strategy of “protest” when they had no fear of retaliatory reactions from the other party. Protesting against the violation of dignity sometimes led to a reduction of psychological pressure for them and sometimes led to a worsening and breakdown of relationships with those around them.

One of the patients stated in this regard:

*“The lab showed my test result as addicted. I protested against the result and became aggressive. One of the nurses in the ward insulted me and said*,* Get lost and do whatever you want. So*,* I went to the last room*,* took out the lamp*,* and harmed myself with it. I know that my behavior is not right and damages my relationships with the nurses*,* but I will prove that I still have the ability to do many things.”* (Patient 1, Male, aged 40).

Patients lost their trust in their families when they observed indifference from them. The family’s neglect of the patients’ requests led to an increase in the patients’ voices of protest.

One of the patients stated:

*“When my mother sees her other children*,* she talks to them and laughs. But she is indifferent to me. She doesn’t value me at all.”* (Patient 12, Male, aged 38).

He continued:

*“This time I was brought to the hospital because of my aggression. Because my mother ignores me*,* I acted out aggressively.”* (Patient 12, Male, aged 38).

#### Mutual disregard for others

Patients express that due to fear of retaliatory actions from those around them, they often do not protest against violations of their dignity and, in such situations, resort to reactions like “Mutual disregard for others”. The disregard following the ignoring of others has dual effects. When patients are forced into silence in the face of dignity violations, this reaction sometimes leads to feelings of resentment due to their inability to defend their rights. At other times, it results in less confrontation with the inappropriate behaviors of those around them and helps maintain the patients’ social image.

One of the patients with schizophrenia says:

*“In response to my family’s disrespect*,* most of the time I don’t respond because of my fear. I remain silent because I want to be at peace. Because if I respond to them*,* they will hit me even harder.”* (Patient 11, Male, aged 41).

Researcher:

*“How do you feel when you stay silent?”*

Patient:

*“I feel lonely and sad*,* and I cry.”* (Patient 11, Male, aged 41).

“Distance from others” was another strategy used by patients to cope with their main concerns.

One of the patients stated:

*“They ignore me*,* and I retreat into my own solitude. Many times*,* I wish I were dead.”* (Patient 12, Male, aged 38).

### Consequence: death wish

Some patients in the study experienced a sense of their dignity being violated by those around them and were severely ostracized by society, they wished for death. This category has two subcategories: “Patient’s internal dissatisfaction” and “Dependency”.

#### Patient’s internal dissatisfaction

Patients who experience a violation of dignity experience greater dissatisfaction in their relationships with family and acquaintances, as well as psychological burnout.

One of the patients said:

“*I had a fight with my mother. Because my mother ignores me and doesn’t listen to me. I’m always alone. I live my life without any purpose. They (my family) ignore me*,* and I retreat into my own solitude*,* which results in zero = zero. Neither do I succeed nor do they succeed. The feeling I have in my life is one of regret. I wish I were dead. Everything is repetitive*.” (Patient 16, Male, aged 30).

Some patients were forced into silence in the face of the violation of their dignity. This reaction sometimes leads to internal tensions in the patient, which causes severe discomfort to the patient. Sometimes, it also leads to fewer fights with those around them and helps maintain the patient’s social status.

One of the patients with schizophrenia says:

“*In response to my family’s disrespect*,* I sometimes reply*,* but most of the time I don’t because I’m afraid. Because if I deal with them reciprocally*,* they will beat me up even more*,* a fight will break out*,* and they will throw me into the hospital*,* and they won’t even visit me and the relationship will get worse. It’s as if I’m not human and don’t deserve respect*.” (Patient 15, Male, aged 22).

Very few patients protested against the violation of their dignity; this reaction sometimes led to a reduction in psychological pressure, while at other times it resulted in the deterioration and breakdown of relationships with those around them.

One of the patients stated in this regard:

“*I got angry at the nurse’s disrespect. I went to the last room*,* took out the lamp*,* and self-harmed with it (there were four 8-centimeter-long scars on each arm due to self-harm). I know that these behaviors are not right and they damage my relationships with the staff*,* but I will prove that I exist*.” (Patient 1, Male, aged 40).

#### Dependency

The complete dependence of the patient on the caregiver becomes an obstacle to maintaining the dignity of the patients. The patient’s inability to perform their duties and personal tasks leads to their devaluation and loss of credibility within the family, resulting in the isolation of patients.

In this regard, one of the patients with schizophrenia says:

“*Because of the stigma of my illness*,* they ignore me. In these circumstances*,* I become withdrawn and can’t appear in society*,* and I become reclusive. In such conditions*,* my illness gradually worsens. No one trusts a person with schizophrenia who is experiencing delusions and hallucinations*.” (Patient 7, Female, aged 46).

According to the experiences of the participants in this study, it can be said that almost all participants have experienced varying degrees of their dignity being trampled and ignored. Overall, the efforts made by the participants to support and protect their dignity were not very effective and had further exposed them to threats to their dignity.

### Core category and theory

In this section, we first explain the main category and then present the theory of “the process of dignity violation in patients with schizophrenia.”

#### The core category: attempting to violate mutual dignity

Patients with schizophrenia were ignored by others. The main concern of these individuals in addressing the violation of their dignity was the act of being ignored by others. When it came to addressing the issue of being ignored by others, patients with schizophrenia utilized the strategy of “Attempting to violate mutual dignity”

The patient expressed:

*“When my brother ignores me*,* I ignore him as well. Just as he values ​​himself*,* I value myself too.”* (Patient 13, Female, aged 39).

#### The theoretical explanation of “Attempting to violate mutual Dignity” in addressing dignity violation in patients with schizophrenia

**Fig. 1:**
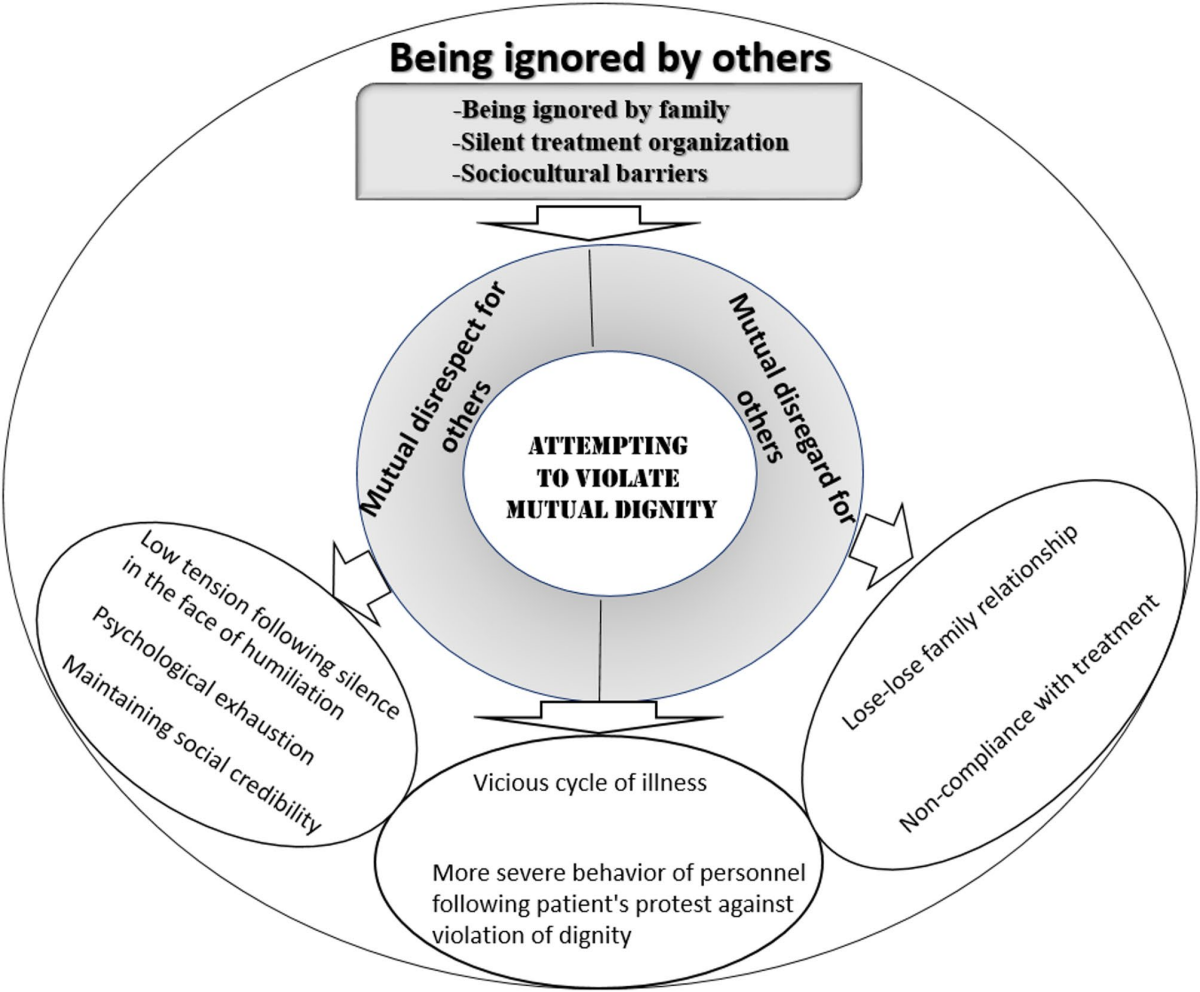
The theory of “Attempting to violate mutual dignity” in addressing dignity violation in patients with schizophrenia

Data analysis showed that the main concern of the patients was “Being ignored by others”. The contextual factors contributing to “Being ignored by others” were “Being ignored by family”, “Silent treatment organization” and “Sociocultural barriers”. What is noticeable in maintaining dignity is that it is formed based on an interaction in which contextual factors are of great importance. Neglect from the family, the medical system, and society prevented them from using their existential capacities and they regressed day by day in life, creating the basis for violating their dignity. Patients used passive strategies such as distancing themselves from those around them and ignoring the humiliation and insults of those around them. Also, in some cases, patients used the protest strategy to express their frustration at the violation of their dignity. Patients used this strategy when they had no fear of the other party’s compensatory reaction. Adopting passive strategies led to mental exhaustion in patients. Ultimately, in such circumstances, they might become hopeless about the future and wish for death. The formation of these negative strategies in patients has led to more negative consequences for them and has further facilitated the violation of their dignity. The theory of “Attempting to Violate Mutual Dignity” in addressing the violation of dignity in individuals with individuals is depicted in Fig. 1.

## Discussion

The present study aimed to explore the process of dignity violation process in patients with schizophrenia. Data analysis revealed that the main concern associated with dignity violation was “Being ignored by others”. The contextual factors of “Being ignored by others” include “Being ignored by family”, “Silent treatment organization” and “Sociocultural barriers”. Structural barriers within the healthcare system, family, and society were identified as factors contributing to the context. The patients expressed that their families exhibit a lack of interest in engaging in communication with them, thereby excluding the patient from their life cycle. Fatigue caused by caregiving challenges causes caregivers to become emotionally distant from their patients. Consequently, to alleviate the strain arising from this shift in the relationship, caregivers endeavor to establish a limited emotional bond [37, 38]. Failing to establish an effective rapport with the patients leads the patients to perceive that their caregivers do not hold them in high regard and disregard their dignity [39].

The results of this study showed that the dignity of patients suffering from mental disorders during their hospitalization, as mentioned in the Bill of Rights of patients, is not considered sufficient. Due to poor human resource management and management inefficiency, a shortage of nursing staff can lead to negligence and indifference in the provision of care and ultimately violation of patient dignity, as shown in previous studies [40–42]. In the psychiatric hospital studied, the care of patients by two nurses and two licensed practical ones made them unable to meet even the basic needs of patients [43]. Respecting the patient’s rights and preventing physical violence are the main aspects of protecting the patient’s dignity [44, 45].

The presence of structural barriers in society is another factor provided the context of “Being ignored by others”. Gostin argues that patients with mental illness often do not have property rights as other citizens [46]. Although the United Nations Convention on the Rights of Persons with Disabilities (CRPD) emphasizes that these patients are entitled to full access to all rights and equal opportunities [19], in reality, they have limited access to health services, particularly in populations with low-income countries. In many countries, there are no community-based rehabilitation programs [20]. The violation of patient rights can therefore lead to a violation of human dignity [22].

What is noticeable in maintaining dignity is that it is based on an interaction and establishes a two-way connection between the patient and those around them. Patients employ the strategy of trying to counteract mutual dignity violations (dominant strategy). One of the passive strategies used by patients in response to dignity violations is mutual disregard. Mutual disregard reflects a non-resistant response from patients when faced with dignity violations, characterized by “indifference towards the humiliation and insults of those around them” and “distancing from those around them”. The concealment of illness and distancing of patients from others in this study is consistent with the modified labeling theory proposed by Link et al. (1989). According to this theory, people could protect themselves from the unfavorable attitudes displayed by others by using a variety of methods, including social distancing and concealment. As predicted by this hypothesis, implementing these tactics may protect patients from some of the negative effects of being labeled. It is crucial to remember, though, that these strategies may also lead to patients becoming socially isolated [47]. In the current study, some patients employed the approach of “silence and indifference toward the humiliation and insults of those around them”. However, this approach is not addressed in the modified labeling theory. The discrepancy can be explained by the fact that the sociocultural setting of the two studies are different; in Iranian culture, for instance, it is valued and respected to be silent, have a passive demeanor, and not react to others’ mistreatment [48]. Remaining silent in the face of being ignored by others causes significant stress and discomfort for patients, and it also deprives them of their rights. The decision to engage in a conflict or not poses a dilemma for patients, as there are interpersonal costs associated with fighting and intra-personal costs associated with not fighting. If they choose to fight, the other party may develop a negative perception of them, whereas if they decide not to fight, they may experience dissatisfaction with themselves at that moment. The choice between fighting and not fighting necessitates a delicate balance between the interpersonal costs of engaging in a conflict and the intrapersonal costs of refraining from doing so [49].

In the present study, in some cases, patients used aggressive strategies and raised their voices to moderate the tensions caused by the indifference of the people around them. The patients said that when they report their problems to their families or nurses and see the indifference of the people around them, their trust in them is lost. Dignity is a Mutual relationship. Hicks states that a person who sees his dignity in danger will be able to violate the dignity of others and to preserve his dignity, he will endanger the dignity of others and perform such behavior that leads to the violation of mutual dignity and Emotional detachment and changing the emotional relationships of the family with the patient [50]. However, according to the patient’s condition, the patient is right. Unfortunately, sometimes, the conflict between the patient and those around him is Mutual. In the present study, the lack of supportive and emotional resources and the ineffectiveness of coping strategies to reduce tensions led to psychological disintegration in patients with schizophrenia. This strategy led to some consequences that have been examined in a main category of “Death wish”. Overall, the efforts that the patients used to protest the threat to their dignity were not effective and further exposed them to threats to their dignity. The category of “Death wish” has two subcategories: “Patient’s internal dissatisfaction” and “Dependency”. The results of the present study showed that the consequences of violating patients’ dignity are widespread and have adverse effects on important aspects of patients’ lives, including employment, marriage, and relationships with others.

Disrespect for human dignity will result in patients may encounter feelings of insecurity, humiliation, and shame. These negative emotions can have a detrimental impact on the outcomes of their treatment and lengthen their stay in the hospital. Additionally, the absence of regard can result in further detrimental health outcomes including apprehension, doubt, shock, loathing, apathy, sadness, and exasperation. Unquestionably, these emotions will have an undeniable effect on the overall health of individuals [23]. On the other, when patients are admitted to the hospital, their dignity can further decline, perpetuating a vicious cycle. Within the hospital environment, patients encounter unfamiliar individuals, must give up their personal and social daily activities and roles, and are expected to comply with tasks assigned to them by the medical staff. These factors contribute to the emergence of new needs and increase the risk of patients losing their dignity [51]. This significantly reduces the motivation of patients and interferes with the progress of their rehabilitation [52]. Jacobs (2001) provided compelling evidence for the integral function of the nurse in advocating for the dignity of patients, positing that the respect for human dignity transcends a mere responsibility of nursing professionals and is fundamentally central to the discipline of nursing, possessing even greater significance than the aspect of health itself [53]. It is incumbent upon psychiatric mental health nurses to uphold and safeguard the respect for the dignity, integrity, and autonomy of the individual [54]. The psychiatric nurses engaged in the provision of patient care encounters challenges and ethical dilemmas on nearly a daily basis. For psychiatric patients who may lack insight into their own physical and psychological needs, it becomes essential for the attending nurses to adhere to established ethical principles while rendering care [55–58]. Within the contextual frameworks of nursing ethical codes, the advocacy for patient autonomy necessitates a meticulous balance with the critical obligation of ensuring patient safety, thereby imposing specific limitations on patient choices in alignment with the ethical principles of beneficence and non-maleficence. The ethical tenets of non-maleficence, deference to patient autonomy, and beneficence represent fundamental elements of nursing codes and standards of practice, which collectively strive to elevate the well-being of patients [59, 60]. It is imperative for nurses to be cognizant of the diverse contentious issues pertaining to psychiatric nursing care and, consequently, to adopt a more proactive stance in safeguarding patients’ rights [61].

## Implication for nursing practice

Mental health nurses play a key role in empowering patients with schizophrenia through problem-solving training to confront dignity violations and reduce feelings of powerlessness. They also engage in combating public stigma by participating in community education and anti-stigma campaigns that promote understanding of schizophrenia. The broader community significantly influences patient dignity by shaping social acceptance or exclusion; therefore, raising awareness and challenging myths at the societal level are crucial to reducing stigma and fostering empathy. Beyond direct care, mental health nurses should address the root causes of dignity violations by fighting stigma and improving public understanding of schizophrenia. Healthcare professionals must create care environments grounded in respect and empathy, continuously addressing negative attitudes through training. Families can also recognize behaviors that lead to social exclusion and undermine the patient’s self-esteem and avoid them, thereby strengthening their role as compassionate caregivers. Collaborative efforts among nurses, families, healthcare providers, and the community are essential to breaking the cycle of dignity violations, promoting social inclusion, and empowering patients to maintain their dignity despite challenges.

## Conclusion

The results of the present study showed that maintaining the dignity of patients with schizophrenia is a downward cycle due to the negative mentality of those around them. Violation of the basic rights of patients by their families, social exclusion, and failure to act according to instructions are common in psychiatric wards. However, the results of this study showed that patients strive to maintain, and regain their dignity even in the most difficult circumstances. Psychiatric nurses can empower patients with the essential abilities to counteract dignity violation, and modify the sense of disability as an obstacle to dignity violation resistance.

### Acknowledgements

This study was part of an extended research project approved and funded by the Vice-Chancellor’s Office for Research at Urmia University of Medical Sciences (IR.UMSU.REC.1401.099). The authors would like to acknowledge financial support for this study and its preparation and/or dissemination. The authors hereby thank all the study participants.

### Strengths and limitations

This study presents a theory about the process of addressing dignity violations, which is new and unique. The designed theory applies to patients, families, and healthcare policymakers. One of the limitations of the present study is that it was short-term. Long-term contact with participants can be a valuable way to identify current and future challenges. Furthermore, this study was conducted in only three cities located in northwestern Iran. Differences in health policies in different regions may provide different insights into the performance of patients and the healthcare system and families. Therefore, we must acknowledge potential bias in sample selection (overreliance on a specific geographical or cultural setting) and limited generalizability of the current findings. However, qualitative studies do not thrive on total facts or accuracy nor eliminate what is considered ‘biases. Bias could be based on preunderstanding, and authors’ preunderstanding affects the interpretive process. The previous knowledge and experience of one of the authors, who worked as a psychiatric nurse in a hospital, was useful for the study, however, had to be treated with caution. critical reflection and discussions among the two authors were employed to help ensure credibility and transferability [36]. 

## Data Availability

The datasets generated and/or analysed during the current study are not publicly available due to privacy and confidentiality concerns, but they are available from the corresponding author upon reasonable request, via Email: [Ebrahimih@tbzmed.ac.ir](mailto: Ebrahimih@tbzmed.ac.ir).
